# The Effects of *Mary Rose* Conservation Treatment on Iron Oxidation Processes and Microbial Communities Contributing to Acid Production in Marine Archaeological Timbers

**DOI:** 10.1371/journal.pone.0084169

**Published:** 2014-02-19

**Authors:** Joanne Preston, Andrew D. Smith, Eleanor J. Schofield, Alan V. Chadwick, Mark A. Jones, Joy E. M. Watts

**Affiliations:** 1 Biological Sciences, University of Portsmouth, Portsmouth, Hants, United Kingdom; 2 STFC Daresbury Laboratory, Warrington, United Kingdom; 3 *Mary Rose* Trust, HM Naval Base, Portsmouth, Hants, United Kingdom; 4 School of Physical Sciences, University of Kent, Canterbury, Kent, United Kingdom; Missouri University of Science and Technology, United States of America

## Abstract

The Tudor warship the *Mary Rose* has reached an important transition point in her conservation. The 19 year long process of spraying with polyethylene glycol (PEG) has been completed (April 29^th^ 2013) and the hull is air drying under tightly controlled conditions. Acidophilic bacteria capable of oxidising iron and sulfur have been previously identified and enriched from unpreserved timbers of the *Mary Rose,* demonstrating that biological pathways of iron and sulfur oxidization existed potentially in this wood, before preservation with PEG. This study was designed to establish if the recycled PEG spray system was a reservoir of microorganisms capable of iron and sulfur oxidization during preservation of the *Mary Rose*. Microbial enrichments derived from PEG impregnated biofilm collected from underneath the *Mary Rose* hull, were examined to better understand the processes of cycling of iron. X-ray absorption spectroscopy was utilised to demonstrate the biological contribution to production of sulfuric acid in the wood. Using molecular microbiological techniques to examine these enrichment cultures, PEG was found to mediate a shift in the microbial community from a co-culture of *Stenotrophomonas* and *Brevunidimonas* sp, to a co-culture of *Stenotrophomonas* and the iron oxidising *Alicyclobacillus* sp. Evidence is presented that PEG is not an inert substance in relation to the redox cycling of iron. This is the first demonstration that solutions of PEG used in the conservation of the *Mary Rose* are promoting the oxidation of ferrous iron in acidic solutions, in which spontaneous abiotic oxidation does not occur in water. Critically, these results suggest PEG mediated redox cycling of iron between valence states in solutions of 75% PEG 200 and 50% PEG 2000 (v/v) at pH 3.0, with serious implications for the future use of PEG as a conservation material of iron rich wooden archaeological artefacts.

## Introduction

King Henry VIII’s Tudor warship the *Mary Rose* provides a unique environment for research into microbial wood degradation, biogeochemical cycling of iron and sulfur and the medium to long-term impacts of conservation of archaeological waterlogged wood. In 1982, after 437 years buried in marine clay sediments off the Southern Coast of England (50°46′N, 1°06′W), the *Mary Rose* was raised from her sea-bed with the aim of restoration, preservation and long term conservation of the ship and artefacts [1]. Typically, challenges faced during conservation of archaeological waterlogged wood include the non-uniform degradation of timbers and the replacement of water within the wood with an inert substance. This must be achieved while avoiding the destructive forces of water that can result in shrinking, warping and cracking of the wooden timbers during the drying process [2]. Water is bound to the hygroscopic cellulose and hemicellulose structural fibres by hydrogen bonding – which once removed causes wood cell shrinkage. In addition, free water occurs throughout the cell lumen providing structural support to the weakened wood cells, but when removed causes collapse [3].

The use of polyethylene glycol (PEG) as a conservation method for wooden shipwrecks was initially developed for conservation of a Swedish warship, *Vasa* [4]. PEG is a polymer of ethylene oxide H(OCH_2_CH_2_)_n_OH [where n = chain length], and demonstrates varying physical and chemical properties at different molecular masses [5]. A two-step preservation method taking advantage of these properties proved successful on the Bremen Cog [6], and it has been applied to the *Mary Rose.* From 1994 until 2006 the Mary Rose was continuously sprayed with an aqueous solution of low molecular weight PEG 200 to penetrate throughout the wood structure. In 2006, the spray was changed to a high molecular weight PEG 2000, which is a waxy solid at room temperature, but soluble in water at elevated temperatures, therefore acting as a consolidant providing structural support by filling voids and lumens of degraded and weakened cells within the wood. The PEG spraying was stopped during April 2013, followed by controlled air drying of the hull.

Compounding the challenges of conservation, waterlogged archaeological wood buried in marine sediments also characteristically accumulate reduced iron and sulfur compounds (RISCs) due to the biogeochemical cycling of Fe and S [7] in anaerobic conditions. Excavation and movement of the *Mary Rose* from an anoxic to an oxic environment therefore posed significant challenges for the conservation of this historic maritime treasure. The acidification of raised marine archaeological timbers and the appearance of sulfate salts on wood surfaces is a degradative phenomenon initially observed in the hull of the Swedish *Vasa* [8], and later in *Mary Rose* artefacts [2], despite conservation efforts with PEG. The oxidation of RISCs upon excavation and the resulting production of sulfuric acid is of major concern to the conservationists. Acid hydrolysis of cellulose and hemicellulose diminishes wood strength via cellular collapse, altering and damaging integral structures. Oxidation of octasulfur (S_8_) to hydrated sulfur salts involves a 5–10x volume expansion per S atom present leading to further mechanical damage at the surface. Iron also has a significant role in this structural damage as Fe^3+^ catalyses the oxidation of sulfides and other reduced sulfur compounds, and direct cellulose degradation by way of a Fenton-type reaction [9].

In a previous study, acidophilic bacteria capable of oxidising iron and sulfur were identified and enriched from unpreserved timbers of the *Mary Rose,* demonstrating that biological pathways of iron and sulfur oxidization exist in archaeological wood before preservation with PEG [10]. One aim of this study was to establish if the recycled PEG spray system is providing a reservoir of potentially harmful iron and/or sulfur oxidising microorganisms, which could be responsible for the accumulation of a substantial biofilm on the barge deck (Figure S1) supporting the *Mary Rose* hull during the conservation process. Due to the non-replaceable nature of the *Mary Rose* experimental model systems were used in this study to simulate known conditions of reduced iron and sulfur compounds in *Mary Rose* timbers. Extant sterile oak pieces containing iron sulfide nano-particles were exposed to microorganisms enriched from *Mary Rose* PEG biofilm samples and analysed using X-ray absorption spectroscopy. We also examined the effect of PEG molecular weight and concentration both on the microbial cultures enriched from the biofilm, and on the abiotic oxidation of iron. This study reports the molecular analysis of the microbial diversity of two *Mary Rose* biofilm derived cultures; an acidophilic iron and sulfur oxidiser enrichment culture maintained at pH 1.7 and associated with acid production in iron sulfide impregnated timbers; and a second less acidic (pH 3.0) enrichment culture containing PEG (BF4-PEG- pH 3), originally initiated from the BF4 pH 1.7 culture. The results from this study have significance to understanding the cycling of iron and sulfur during conservation of the *Mary Rose* hull and other archaeological treasures.

## Methods

### Enrichment Cultures, PEG Molecular Weight and Concentration Series

The *Mary Rose* has been stored at a custom built facility since 1994, with continual spraying with PEG 2000 at 28°C; the concentration was slowly increased to 70% over time. The PEG was applied by a recycling spray system, and as a result a 1–4 cm rust coloured PEG impregnated biofilm accumulated on the barge deck supporting the *Mary Rose* ship, but, not directly upon the *Mary Rose* structure (Figure S1). The biofilm was sampled using aseptic technique from three locations around the barge. The biofilm samples were combined and 1 g was resuspended and vortexed in 9 mls sterile PBS, this was the inoculum for cultures used to enrich for acidophilic and chemolithotrophic iron and sulfur oxidisers using liquid and solid agarose media as previously described [10], [11]. Minimal liquid media contained 0.4 g/L MgSO_4_.7H_2_O, 0.2 g/L (NH_4_)_2_SO_4_, 0.1 g/L K_2_HPO_4_ with or without 10 g/L NaCl. Medium was adjusted to pH 1.7 or 3.0 with H_2_SO_4_. Growth substrates included 25 mM Fe(II)SO_4_, 2.5 mM K_2_S_4_O_6_, elemental sulfur 5 g/L or pyrite (iron sulfide) and incubated at 22°C for 14 days. After 5 sequential transfers to enable enrichment of the iron sulfur oxidisers the enrichment cultures were used to inoculate minimal media containing PEG 200 (0%, 25%, 50%, 75%) and PEG 2000 (0%, 12.5%, 25%) with iron sulfide impregnated wood blocks that were subsequently analysed by X-ray absorption spectroscopy.

### Abiotic Iron Oxidation Trials at Different Concentrations of PEG 200 and PEG 2000

To test for an abiotic effect of PEG on iron (II) oxidation, a range of concentrations of PEG 200 (0%, 25%, 50%, 75%) and PEG 2000 (0%, 12.5%, 25%) solutions were diluted in minimal media (as described above) containing 25 mM Fe(II)SO_4_. pH 1.7 Iron oxidation was measured using a colorimetric Ferrozine assay as previously described [12]; both concentration series of PEG were tested at pH 3.0 and pH 6.0, in triplicate. Stationary 250 mL conical flasks were used containing 100 ml of the minimal media and source of reduced iron, held at 21°C and hand mixed every 12 hours, when assays were performed. Measurements of pH were taken, using Unisense electrodes (Unisense, Denmark). The flasks were observed after inoculation with iron and measurements were taken after the first hour and there after every 12 to 24 hours.

### Impregnating Oak Blocks with Iron Sulfide

Due to the restricted availability of original *Mary Rose* wood, to replicate the distribution of nano-particles of iron sulfide distributed throughout *Mary Rose* archaeological wood [8], extant sterile English Oak was impregnated with iron sulfides and subsequently used in enrichment cultures derived from the *Mary Rose* biofilm. This approach has been used by others previously to examine the microbes involved in the degradation of the *Vasa* shipwreck [7]. Fresh blocks of oak approx 25 mm x 25 mm x 5 mm were cut by diamond saw, both transversely and longitudinally to the wood vessels. The wooden blocks were impregnated with iron sulfates by soaking the oak blocks in 0.1 M sterile Fe(II)SO_4_ solution at 18°C for 4 days followed by passing hydrogen sulfide gas through the solution for 2 days. The iron sulfide impregnated blocks were rinsed in sterile water before immersion in sterile media inoculated with 1 ml of enrichment culture generated from the PEG impregnated biofilm for 21 days at 22°C under aerobic conditions. Controls were set up in the same way using the iron sulfide impregnated oak blocks in sterile media.

### XANES Absorption Spectroscopy Analysis

The speciation state of iron and sulfur in the oak was analysed by X-ray absorption spectroscopy at Diamond Light Source, using the microfocus EXAFS beamline I18 [13]. Thin transverse sections of wood were cut by hand from the wood blocks with a razor blade and mounted between sheets of Ultralene™ film which kept them hydrated during data collection. The speciation of the sulfur and iron compounds was determined by sulfur and iron K-edge X-ray Absorption Near Edge Structure (XANES) spectroscopy, with energy calibrations determined from the inflection point of the rising edge of elemental reference materials. A metal iron foil was used to calibrate the Fe^0^ edge at 7112 eV. For the sulfur, the edge position was calibrated to 2472 eV for S^0^ using elemental sulfur, with shifts in edge position expected due to sulfur valency [14]. Qualitative analysis performed by comparing the data to a library of known iron and sulfur compound standards, a common ‘fingerprinting’ technique used to determine shifts in oxidation state. Standard compounds used to identify the key iron valence states were iron sulfide, Fe(II)SO_4_, Fe(III)SO_4_ and Fe_2_O_3_, with edge positions measured at 7118.8, 7125.2eV, 7128.8 and 7126.5 respectively. For sulfur the edge position was calibrated to 2472 eV for S^0^ using elemental sulfur.

### Molecular Analysis of Microbial Diversity

DNA extractions of the microbial communities present within enrichment cultures were performed using PowerSoil DNA extraction kits (MOBIO Laboratories, Inc. USA). The 16S ribosomal RNA genes were amplified using a nested PCR approach, with primer pairs 27f/1492r and 357f/1392r [15], [16]. PCR reactions were performed in triplicate and pooled for each DNA extraction. Despite the limitations, this approach was necessary due to the inhibitory effects of high concentrations of iron and the low cell yield numbers typical of acidophilic iron and sulfur oxidising cultures [17]. The PCR was performed using Hi-Fidelity PCR Master Mix 1 (Thermo Scientific product AB-0792) with the addition of 0.2 µM forward and reverse primers and 5–10 µg/mL genomic DNA. The 27f/1492r PCR cycle conditions were as follows: initial denaturation of 5 mins at 94°C; 35 cycles of 60 sec at 94°C, 60s at 59°C, 60s at 72°C; followed by a final extension step of 10 mins at 72°C. The PCR product was purified with Qiagen™ QIAquick® PCR purification kit and quantified. The 357f/1392r fragment amplification PCR was performed as detailed before except with 0.1 µM forward and reverse primers and 1 µg/mL of the purified 27f/1492r PCR amplicon. The 357f/1392r PCR cycle conditions were, initial denaturation of 5 mins at 94°C; 30 cycles of 30 secs at 94°C, 30s at 59°C, 30s at 72°C, followed by a final extension step of 10 mins at 72°C then held at 4°C. Cloning and transformation was performed using TOPO TA cloning® kit (Invitrogen™). A total of 96 clones were selected from each enrichment culture clone library, plasmid purified and sequenced (Functional Biosciences, Inc™, USA). Contigs were assembled using the CAP3 [18] and checked for chimeric sequences using Bellerophon within Greengenes [19] and used as search queries on BLAST [20]. Sequences were aligned using the ClustalW alignment algorithm. All phylogenetic analysis was performed in MEGA 5.05 [21] and sequences deposited in GenBank (KC249982–KC249994).

## Results

### XANES Analysis of Iron Sulfide Impregnated Wood Exposed to *Mary Rose* Microbial Enrichment Cultures

**Figure 1:**
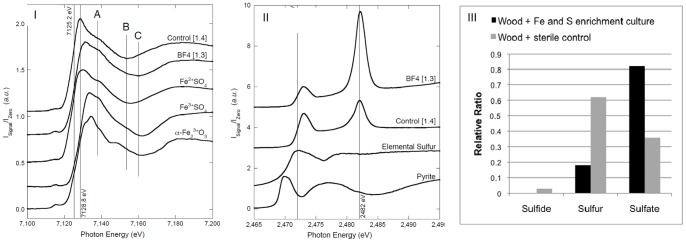
XANES analysis of wood blocks impregnated with FeS_2_. The oak blocks were exposed to Fe and S acidophilic culture (BF4- pH 1.7) enriched from biofilm accumulating on the *Mary Rose* barge deck during PEG spraying treatment, and a sterile media control (Control). Panel I: Fe K-edge XANES spectra of wood samples compared to reference spectra from ferrous sulfate, ferric sulfate and iron oxide. Panel II: S K-edge XANES analysis of wood samples compared to reference spectra from elemental sulfur and fresh pyrite. Panel III: Relative ratios of sulfur compounds in wood samples.

The oak blocks were removed from the enrichment culture BF4 pH 1.7 and the abiotic control containing sterile media. A biofilm was present (observed by light microscope) on the surface of the wood exposed to enrichment culture BF4 pH 1.7, but, absent on the sterile control. A thin section was taken from the surface of the wood and several X-ray absorption near-edge structure (XANES) iron and sulfur spectra were collected from different locations for each sample. XANES spectra for the iron and sulfur from both biofilm and control specimens along with reference compounds are given in Figure 1, Panel I and Panel II respectively. Spectra were normalised to give a constant edge jump and are shown, offset for clarity. XANES Fe *K*-edge spectra were collected at several locations from the BF4 pH 1.7 enrichment culture wood (3 locations) and abiotic control (5 locations) and a representative spectrum is shown in Figure 1 (Panel I). The Fe K-edge position shows a small shift from 7125.3 eV for the control, to 7125.8 eV for enrichment culture BF4 pH 1.7. This is in agreement with the iron (II) valence obtained from the ferrous sulfate reference and suggests that the BF4 pH 1.7 enrichment culture is having a small oxidising effect on the iron component in the timber.

Sulfur XANES spectra were also collected from several locations on each sample (5 locations for BF4, 2 locations in the control) and averaged to produce the spectra shown in Figure 1 (Panel II). A sulfur peak is observed at 2473 V, representative of sulfur species commonly found in historic marine timber [8], [14]. The spectra also show the S^+6^ sulfate peak at 2482 eV, which is much more prominent for the BF4 pH 1.7 sample than the control, clearly demonstrating the sulfur oxidising behaviour of the microbial enrichment culture BF4 pH 1.7.

### Biotic Iron Oxidation Trials at Different Concentrations of PEG 200 and PEG 2000

To examine the interaction of microbial communities with PEG, the initial enrichment culture BF4 pH 1.7 was used to inoculate a range of media containing PEG 200 and PEG 2000 at different concentrations. At pH 3 rapid oxidation of iron (II) was observed in media containing PEG 200 75% concurrent with a change in colour from transparent to rust orange with substantial iron (III) oxide deposits on the flask surface. This rapid iron oxidising culture, BF4-PEG- pH 3, was compared with its original inoculum (culture BF4 pH 1.7) using molecular analysis, to examine any changes in the microbial diversity.

### Clone Libraries and Phylogenetic Analysis of BF4 pH 1.7 and BF4-PEG- pH 3

**Figure 2:**
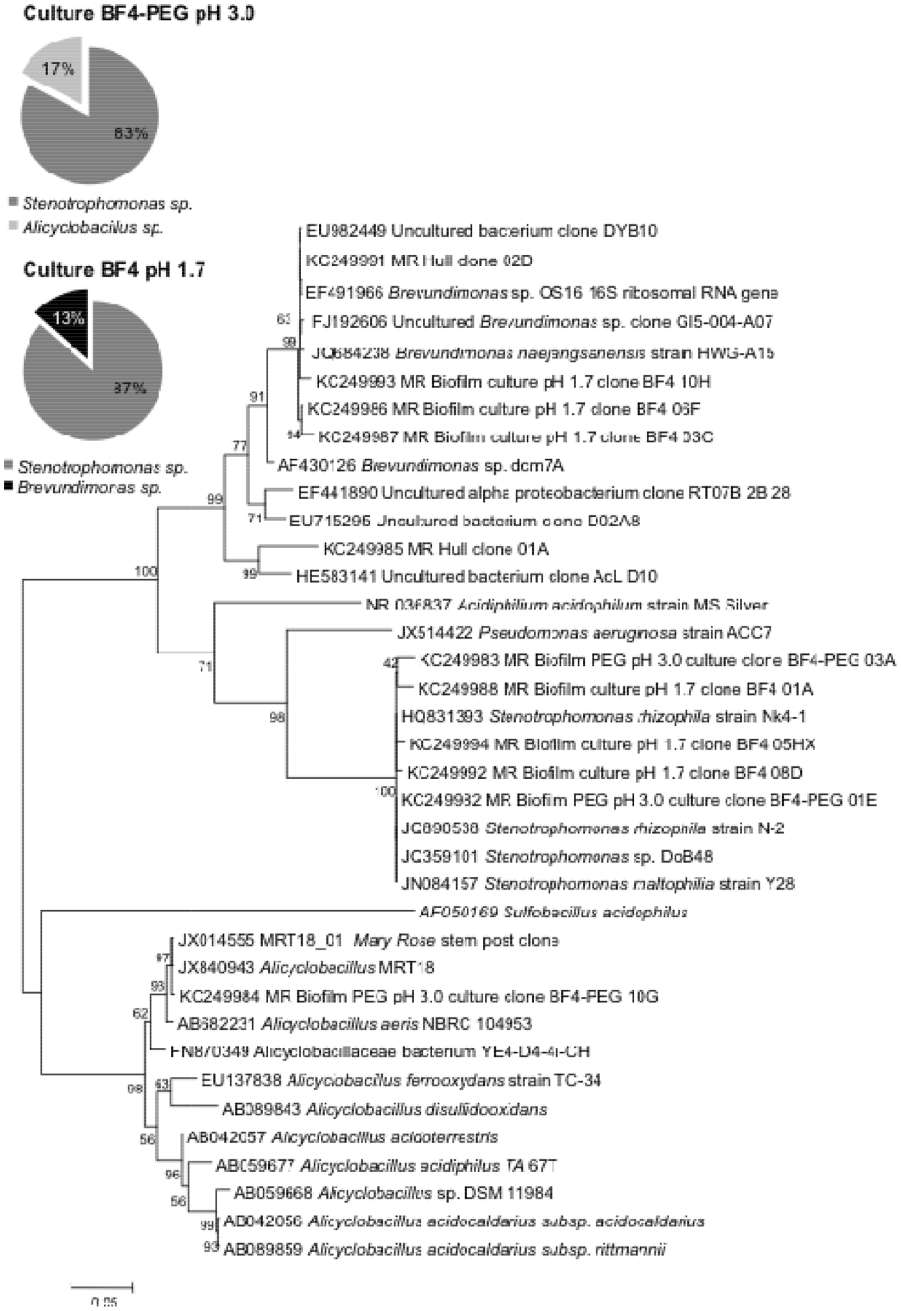
Maximum Likelihood phylogenetic tree of 16S ribosomal RNA gene sequences from the *Mary Rose* biofilm enrichment cultures BF4-low pH and BF4-PEG with related outgroup taxa. Substitution pattern and rates were estimated under the General Time Reversible model (+G), where gamma distribution is estimated at 0.3005. The nucleotide frequencies are A = 20.34%, T/U = 24.84%, C = 32.08%, and G = 22.74%. For estimating ML values, a user-specified topology was used. The maximum Log likelihood for this computation was -6907.774. The analysis involved 42 nucleotide sequences. All positions containing gaps and missing data were eliminated. There were a total of 978 positions in the final dataset. Evolutionary analyses were conducted in MEGA5 [2]. Pie charts display bacterial diversity of *Mary Rose* biofilm enriched in minimal media at pH 1.7 (BF4-pH 1.7) and BF4-PEG-pH 3.0 enrichment culture with 75% PEG 200.

From the XANES analysis a microbially mediated effect on speciation of iron and sulfur in oak timbers was detected in the BF4 pH 1.7 enrichment culture compared to the sterile media control. To examine the microbial diversity of this acidophilic enrichment culture (BF4 pH 1.7) 16S rRNA gene analysis was performed. From this molecular analysis there were 3 distinct groups of acid tolerant or acidophilic bacteria represented in the clone libraries from the *Gammaproteobacteria*, *Alphaproteobacteria* and *Firmicutes* (Figure 2). A total of 90 clones were successfully sequenced and assembled from the biofilm enrichment culture (6 potential chimeric sequences removed from the analysis) BF4 pH 1.7, revealing a bacterial co-culture of gammaproteobacteria and alphaproteobacteria (Table 1). Based on 16S rRNA gene sequence similarity BLAST searches, the dominant taxa (85% of clone library) shared 99% similarity to known *Stenotrophomonas rhizophila* and *S. maltophililia* strains represented by clones BF4-01A, BF4-05H, BF4-08D (Figure 2). The remaining 15% of clone library sequences were of 99% similarity to known and uncultured *Brevundimonas* sp. The alphaproteobacterial *Brevunidmonas* clade presented in the phylogenetic tree in Figure 2, contains sequences from both the BF4 pH 1.7 culture, represented by clones BF4-03C, BF4-06F and BF4-10H and sequences previously detected in the *Mary Rose* wooden hull without enrichment, represented by clones MR Hull -02D (KC249991), [10] . These sequences shared highest similarity to *Brevundimonas* strain OS16 [22] forming a distinct clade with 99% bootstrap support. When the BF4 pH 1.7 acidophilic enrichment culture was used to inoculate the same basic medium adjusted to pH 3.0, but containing 75% PEG 200 (v/v), rapid iron (II) oxidation was observed concurrently with a shift in clone library composition. A total of 91 clones (5 chimeric sequences were removed from the analysis) were successfully sequenced from the BF4-PEG- pH 3 enrichment culture, revealing another acid tolerant bacterial co-culture of γ-proteobacteria and Firmicutes. The gammaproteobacteria *Stenotrophomonas* sp. remained the dominant taxa in the BF4-PEG- pH 3 clone library (81% of sequences), represented by clones BF4-PEG–01E and BF4-PEG-03A and sharing a clade with the BF4 pH 1.7 *Stenotrophomonas sp.* with 100% bootstrap support (Figure 2). However, the *Brevundimonas* found at the lower pH were replaced by *Alicyclobacillus*-like sequences; clone BF4-PEG-10G is of high similarity (99.7%) to *Mary Rose Alicyclobacillus* clone MRT18-01 (and cultured strain MRT18) (JX014555) previously isolated from the unpreserved *Mary Rose* stem post [10], and formed a monophyletic clade (97% bootstrap support), see Figure 2. The obligate halophillic, facultative chemolithotrophic iron oxidising *Alicyclobacillus* strain MRT-18 is a spore forming bacteria that requires a minimum of 1% NaCl for growth.

**Table 1:** *Mary Rose* Hull and Biofilm acidophile enrichment culture clone library diversity.

Clone name and GenBankAc. No.	% clones	Highest BLAST search similarity andGenBank Ac. No.	% similarity	Bacterial phylum/class
**Mary Rose Biofilm culture BF4-PEG-pH 3 (81 clones)**				
BF4-PEG 01E (KC249982)	80	*Stenotrophomonas rhizophila* (JQ890538)	99	γ-proteobacteria
BF4-PEG 03A (KC249983)	3	*Stenotrophomonas rhizophila* (JQ890538)	98	γ-proteobacteria
BF4-PEG 10G (KC249984)	17	*Alicyclobacillus* sp. MRT18 (JX840943)	99	Bacilli
**Mary Rose Biofilm culture BF4-pH 1.7 (90 clones)**				
BF4 06F (KC249986)	1	*Brevundimonas* sp. OS16 (EF491966)	98	α-proteobacteria
BF4 03C (KC249987)	1	*Brevundimonas* sp. OS16 (EF491966)	98	α-proteobacteria
BF4 10H (KC249993)	11	*Brevundimonas* sp. OS16 (EF491966)	99	α-proteobacteria
BF4 01A (KC249988)	5	*Stenotrophomonas rhizophila* (JQ890538)	98	γ-proteobacteria
BF4 08D (KC249992)	12	*Stenotrophomonas rhizophila* (JQ890538)	99	γ-proteobacteria
BF4 05HX (KC249994)	70	*Stenotrophomonas rhizophila* (JQ890538)	99	γ-proteobacteria
**Mary Rose Hull 5 mm wood core**				
MR Hull 01A (KC249985)	1	*Brevundimonas* sp. dcm7A - (AF430126)	90	β-proteobacteria
MR Hull 02D (KC249991)	3	*Brevundimonas* sp. OS16 (EF491966)	99	α-proteobacteria

Clone library sequence classification and diversity of representative OTUs defined at 97% or greater similarity. Taxonomic classification according to Ribosome Database Project Classifier tool (Wang et al. 2007) and Blast search results with GenBank Accession number of top hit (in parenthesis). The percentage of clones is given for each OTU. The Mary Rose Hull core clones are from a clone library containing 80 clones (submitted to GenBank), of which 4 are listed here as the sequences are included in the phylogenetic clade containing *Brevundimonas* biofilm culture clones.

### Abiotic Iron Oxidation Trials at Different Concentrations of PEG 200 and PEG 2000

**Figure 3:**
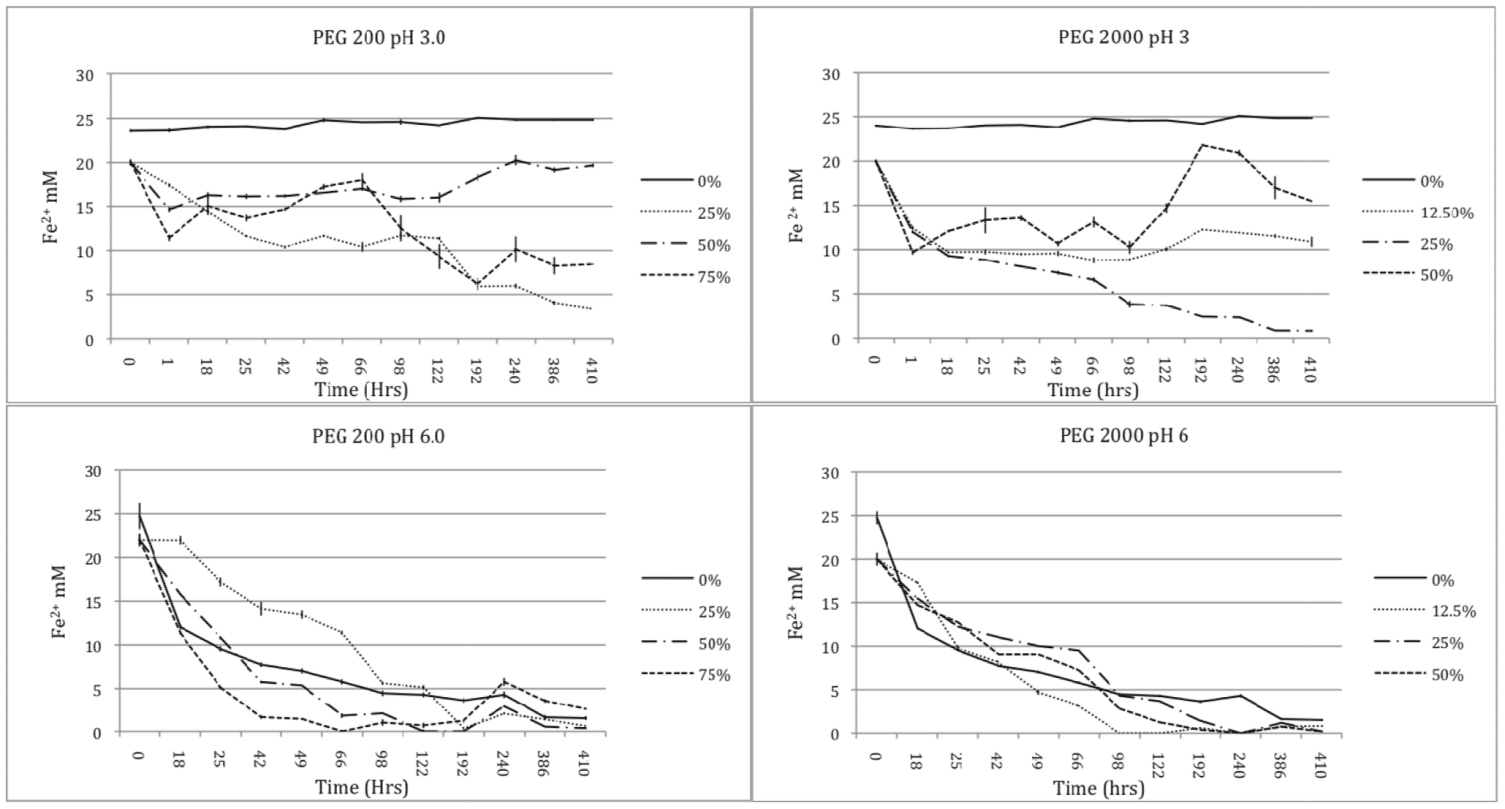
Oxidation rates of ferrous iron in a range of PEG 200 and PEG 2000 concentrations at pH 3.0 and pH 6.0 pH values.

The iron oxidation trials with different concentrations of high and low molecular weight PEG suggest that the addition of this polymer altered the redox state of iron at pH 3, compared with controls containing no PEG (Figures 1 and 3). At pH 3, addition of PEG 200 and 2000 altered oxidation state in comparison to the no addition control. At pH 6 these effects were minimised in both PEG 200 and 2000, possibly due to the dominant abiotic oxidation of iron (II). As shown in Figure 3, the relationship between pH and iron (II) oxidation rates in minimal media without PEG is very clear, abiotic oxidation of iron (II) is observed at pH >4. The colorimetric ferrozine assay provides a robust tool to analyse the behaviour of iron, but loses sensitivity at the lower concentrations due to lack of spectroscopy detection.

In the 0% PEG solution at pH 3.0 the iron (II) concentration remained constant, but, decreased in the 0% PEG solution at pH 6.0, as expected, due to abiotically driven chemical oxidation of the iron (II). Also as expected at pH 6.0 the iron (II) was completely oxidised regardless of PEG concentration or molecular weight (Figure 3), however, the 25% PEG 200 solution delayed the oxidation of iron (II) by approximately 18 hrs and exhibited a slower rate of oxidation than the 0%, 50% and 75% PEG 200. Furthermore, in both the PEG 200 and PEG 2000 solutions there was a concentration dependent effect on iron (II) oxidation at the lower pH 3.0 compared to the 0% PEG free sterile media controls (Figure 3). At 0% PEG there was no spontaneous abiotic oxidation of iron (II) at pH 3.0 for more than 400 hrs. In the PEG 200 series, there was near complete oxidation of the iron (II) in the 25% PEG 200 solution (<3.5 mM Fe^2+^). In contrast, the 50% PEG 200 solution exhibited some initial reduction in iron (II) concentration, but, little overall decrease in iron (II), for the duration of the experiment, ferrous iron concentration remaining at between 15–20 mM (>400 hrs).

Critically for iron present in solution, PEG 200 75% caused the cyclic change between oxidised and reduced states, an initial decrease in iron (II) was followed by a gradual increase to approx 18 mM after 66 hrs, followed by a decrease in iron (II) to 6 mM and concurrent precipitation of iron (III) particles in the solution. These effects were mirrored in the higher molecular weight PEG 2000 concentration series. The 50% PEG 2000 solution exhibited a similar, but, stronger cycle of iron (II) oxidation concurrent with a colorimetric change from transparent to rust orange solution, with iron (III) precipitation followed by a rapid increase in iron (II) concentration to starting levels and a return of the solution to transparent as the iron (III) was subsequently reduced. At 25% PEG 2000 pH 3.0, the iron (II) concentration reduced with almost total oxidation to the ferric state, and the iron (II) in the 12.5% PEG 2000 pH 3.0 solution was partially oxidised, but, remained at approximately 10 mM after 18 hours to the end of the experiment. This concentration dependent effect of PEG on iron redox cycling cannot be explained simply by a buffering effect, since there is no correlation between iron (II) concentration/oxidation state and the final pH of the solution in the pH 3.0 PEG series. The mechanism of the concentration dependent redox cycling of iron therefore remains unknown, but appears to be critically important in relation to its use as an inert consolidant.

## Discussion

The study of the biological oxidation of iron presents several challenges. The redox potential of the iron (II)/(III) couple at low pH (+0.77 V) means iron oxidation is only thermodynamically feasible with O_2_ as an electron acceptor. Anaerobic iron oxidation mediated by nitrate-reducing bacteria and via anoxygenic photosynthesis can occur, but only at neutral pH [23]. Consequently the large amount of iron (II) required to sustain growth means there is a typically low cell yield in cultures of acidophilic iron oxidising chemolithotrophs. The challenges are compounded when investigating an environment such as the *Mary Rose* with a highly variable distribution of iron and sulfur compounds, heterogeneity of pH across the timbers, and limitations of sampling due to the historical and archaeological value of the artefact. Furthermore, the biological contribution to the oxidation of iron (II) at low pH is a difficult system to measure due to the chemical pathways involved. Abiotic pyrite oxidation by iron (III) ions at low pH [24] potentially removes the iron (III) generated by iron oxidising acidophiles, by reducing it to iron (II) as long as accessible iron sulfide exists in the wood to drive this abiotic pathway. However, despite these limitations, this research presents the successful application of XANES to further our understanding of the microbial contribution to iron and sulfur oxidation in archaeological preservation.

The results from the sulfur and iron K-edge XANES experiment demonstrates that microorganisms enriched from the *Mary Rose* biofilm are mediating a shift to the oxidised states of iron and sulfur in oak impregnated with reduced iron sulfide compounds. There is XANES evidence to support biological acid production from the oxidation of iron sulfides and reduced sulfur compounds in wood to sulfates. These changes were mediated by organisms present in culture BF4 pH 1.7 enriched from a PEG impregnated biofilm taken from the *Mary Rose* barge deck. Importantly, these results show that the bacterial consortia enriched from PEG impregnated *Mary Rose* biofilm are capable of oxidation of iron sulfide to sulfate over a relatively short period of time (21 days). There is also a shift from the iron (II) to the iron (III) oxidation states in oak timbers immersed in the BF4 pH 1.7 culture, indicating that biological iron (II) oxidation is also occurring. The iron and sulfur compounds in the control samples remain unchanged, however, reflecting the lack of chemical oxidation of iron (II) at low pH.

Although we cannot assign function to the organisms detected in the co-cultures without additional studies, a number of phylotypes are identified that may play a role in the oxidation of the iron and sulfur compounds in the wood. The proteobacterial species identified in the BF4 pH 1.7 clone library are closely related to initial biofilm formers; such as *Alphaproteobacteria* that are known to utilise the polysaccharides from extracellular polymeric substances (EPS) that stabilise biofilms as a carbon source for growth [25]. The *Stenotrophomonas* is a highly adaptable genus [26] commonly found in soils and plants; *S. maltophilia* is involved in the formation of biofilms adhesion to surfaces [27]. Interestingly, a previous report found using culture based studies that *Stenotrophomonas spp*. were indicated as PEG/PPG utilizing bacterium via a Fenton-type reaction in the presence of a reductant and iron (III) [28]. However, further studies must test the involvement of the *Stenotrophomonas spp.* as they have been described as common contaminants of PCR reactions [29].

The acid tolerant *Brevundimonas* bacteria identified in culture BF4 pH 1.7 is closely related to a *Brevundimonas* strain isolated from Zloty Gold mine rocks containing high levels of iron sulfides, and known to possess the iron-binding sideophores that play an important role in iron uptake from insoluble minerals [22]. Figure 2 demonstrates that this clade was also closely related to uncultured alphaproteobacterium clones isolated from the Rio Tinto [25], [30]. Further work is required to identify and characterise the species responsible for sulfur oxidation at low pH in culture BF4 pH 1.7. It is however, highly relevant that the same species of *Brevundimonas* present in culture BF4 pH 1.7 is also present in clone libraries from wood cores of the *Mary Rose* hull, indicating its presence in the co-culture may be functionally related to the oxidation of iron or sulfur in archaeological wood.

This study presents evidence that PEG is not an inert substance in relation to the redox cycling of iron. Furthermore, the addition of PEG to chemolithotrophic minimal media results in a functionally significant shift in microbial community structure. The community shift after 75% PEG 200 (v/v) addition appears to alter the conditions to better support the iron oxidising *Alicyclobacillus* bacteria rather than the *Brevundimonas* spp. The iron oxidising *Alicyclobacillus* bacteria observed in BF4-PEG- pH 3, were either previously dormant (*Alicyclobacillus* MRT18 is a known spore forming bacteria) or present in very low numbers. The PEG may provide a carbon source complemented by the decreased acidity (pH increased from pH 1.7 to pH 3.0) that enabled the growth of the iron oxidising *Alicyclobacillus* species. The immersion of iron sulfide impregnated oak blocks in the acidic media (pH 1.7) used in the synchrotron experiment may have created microniches of higher pH, extant oak having a natural acidity of pH 3.0–3.8. This would not have facilitated the abiotic oxidation of iron (II), but may have enabled the growth of the less extremely acidophilic *Alicyclobacillus* sp. and consequently the biological oxidation of iron (II) observed in the XANES analysis of the oak blocks. It is of significance to the conservation process that the PEG compound used to stabilise the *Mary Rose* hull may provide a carbon source for the microbial oxidation of reduced iron and sulfur compounds by acidophilic bacteria in the archaeological timbers.

Abiotic iron redox cycling was also observed in the acidic media solutions (pH 3) of PEG 200 and PEG 2000. These results strongly indicate that PEG has concentration dependent properties that catalyse the redox cycling of iron, independent of microbial activity, in low pH solutions where iron (II) remains relatively stable. Previously, it has been demonstrated that at higher pH (pH 6), PEG 400 -water solutions have a concentration dependent effect on the rate of iron corrosion, driven by an evolution of pH and consequent increase or decrease in conductivity, dissolved oxygen solubility and complexation of iron ions by PEG [31]. This is the first demonstration that both low and high molecular weight solutions of PEG used in the conservation of the *Mary Rose*, (PEG 200 and PEG 2000) are promoting the oxidation of ferrous iron in acidic solutions at which spontaneous abiotic oxidation does not occur in water. These results are of relevance to the suitability of PEG as an inert material for conservation of marine archaeological timbers containing reduced forms of iron, the major concern being that PEG can facilitate the corrosion of iron sulfides, and catalyse the consequent sulfuric acid production. This is compounded by the observation that the PEG appears to promote the growth of heterotrophic iron oxidising acidophilic bacteria in low pH environments.

This report demonstrates the value of combining molecular analysis of microbial diversity with culture dependent model systems and applying an interdisciplinary approach to unravel the complex processes of iron sulfide oxidation in archaeological wood at low pH values. Microbial cultures derived from the *Mary Rose,* enriching for acidophilic sulfur- and iron- oxidizing consortia have provided useful tools, which were subsequently applied to a model system to test for *in situ* biological oxidation of reduced iron and sulfur compounds in oak wood using X-ray spectroscopy. The detection of sulfates from biotic oxidation of iron sulfide clearly demonstrates the significance of microbial communities to the ‘sulfur problem’ in marine archaeological wood, and consequent degradation of historically important maritime treasures. Further research into the abiotic mechanisms of PEG dependent iron corrosion in acidic environments is essential if we are to fully understand the complex interaction of abiotic and biotic pathways of iron sulfide oxidation at low pH. This study has serious implications for the future use of PEG as a conservation material of iron rich archaeological artefacts. Based on this research, a combined approach of effective biocidal treatment combined with the removal or inactivation of reduced iron and sulfur compounds, is recommended before PEG is considered as an appropriate long-term conservation material for archaeological waterlogged wood/artefacts.

## Supporting Information

Figure S1 **The** ***Mary Rose*** **undergoing PEG treatment.** PEG spray was continuously recirculated and arrow indicates the location of the barge deck. Image provided by the Mary Rose Trust.
(TIF)
