# Supplementary figures and images for: The Effects of Mary Rose Conservation Treatment on Iron Oxidation Processes and Microbial Communities Contributing to Acid Production in Marine Archaeological Timbers
Source: PLoS One. 2014 Feb 19;9(2):e84169. doi: 10.1371/journal.pone.0084169 (PMC3929279; doi:10.1371/journal.pone.0084169)

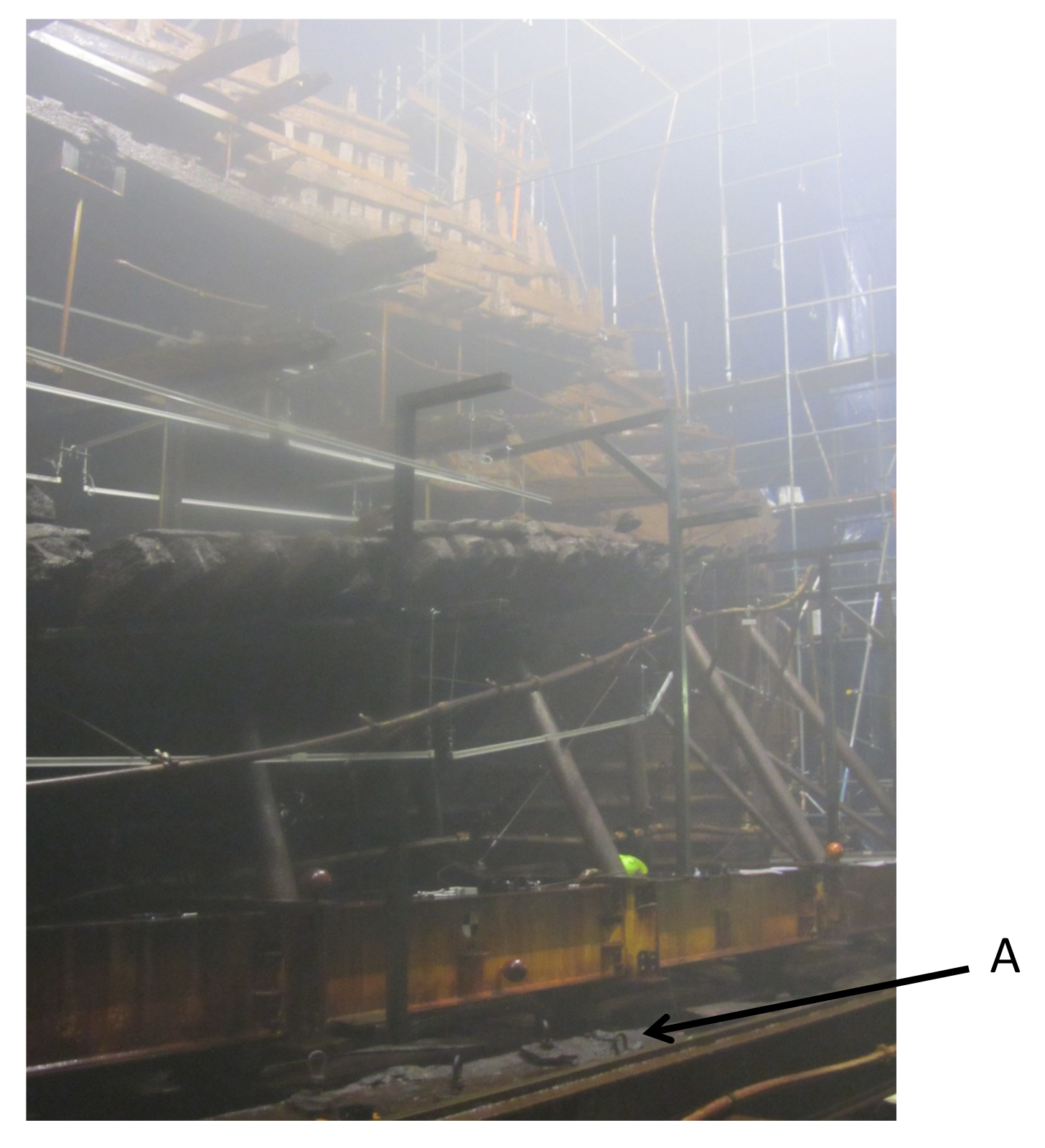

Supplement: Figure S1 — The Mary Rose undergoing PEG treatment. PEG spray was continuously recirculated and arrow indicates the location of the barge deck. Image provided by the Mary Rose Trust. (TIF) [file pone.0084169.s001.tif]
